# Effect of Carbodiimide (EDC) on the Bond Strength Longevity of Epoxy Resin-based Endodontic Sealer to Root Dentin: An In-Vitro Study

**DOI:** 10.3290/j.jad.b4097187

**Published:** 2023-05-16

**Authors:** Pedro Henrique Bernardes Caetano, Helena Cristina de Assis, Leonardo Moreira Teodosio, Gunther Ricardo Bertolini, Renato Roperto, Manoel Damião Sousa-Neto, Fabiane Carneiro Lopes-Olhê

**Affiliations:** a Graduate Student, Department of Restorative Dentistry, School of Dentistry of Ribeirão Preto, University of São Paulo (USP), Ribeirão Preto, São Paulo, Brazil. Performed the endodontic treatment and data acquisition, wrote the manuscript, approved the version to be submitted.; b Postgraduate Student, Department of Restorative Dentistry, School of Dentistry of Ribeirão Preto, University of São Paulo (USP), Ribeirão Preto, São Paulo, Brazil. Substantial contributions to the conception and design of the study. Statistical analysis and interpretation of results, approved the version to be submitted.; c Postgraduate Student, Department of Restorative Dentistry, School of Dentistry of Ribeirão Preto, University of São Paulo (USP), Ribeirão Preto, São Paulo, Brazil. Data acquisition and analysis, critically revised the manuscript, approved the version to be submitted.; d Postgraduate student, Department of Restorative Dentistry, School of Dentistry of Ribeirão Preto, University of São Paulo (USP), Ribeirão Preto, São Paulo, Brazil. Wrote the manuscript and approved the version to be submitted.; e Professor, Department of Adult Restorative Dentistry, University of Nebraska Medical Center, UNMC College of Dentistry, Lincoln, NE, USA. Substantially contributed to the study idea, critically revised the manucript and approved the version to be submitted.; f Professor, Department of Restorative Dentistry, School of Dentistry of Ribeirão Preto, University of São Paulo (USP), Ribeirão Preto, São Paulo, Brazil. Study idea, critically revised the manucript and approved the version to be submitted.; g Professor, Department of Restorative Dentistry, School of Dentistry of Ribeirão Preto, University of São Paulo (USP), Ribeirão Preto, São Paulo, Brazil. Study idea, substantially contributed to the data analysis and interpretation, co-wrote the manuscript.

**Keywords:** carbodiimide, EDTA, dentin, root canal filling.

## Abstract

**Purpose:**

EDC (1-ethyl-3-(3-dimethylaminopropyl)carbodiimide hydrochloride) can increase dentin bonding longevity. This study aimed to evaluate the effect of final irrigation of the root canal with EDC on the bond strength (BS) longevity of an epoxy resin-based root-canal sealer.

**Materials and Methods:**

Twenty maxillary canines were sectioned and standardized for root length at 17 mm. Roots were instrumented and distributed into 2 groups according to the final irrigation protocol: EDTA 17%+NaOCl 2.5% (C) and EDTA 17%+NaOCl 2.5%+EDC 0.5M (EDC). The canals were dried and filled with AH Plus (Dentsply Sirona). Three slices were obtained per third, and the first slice from each third was used for the immediate push-out test (i) followed by analysis of the failure pattern (n = 10); the second slice from each third was used for the push-out test after 6-month aging (A) followed by analysis of the failure pattern (n = 10); the third slice from each third was used to examine the adhesive interface under confocal laser scanning microscopy (CLSM) (n = 10). Data were analyzed with ANOVA, Fisher’s exact and Kruskal-Wallis tests.

**Results:**

Higher BSs were found for EDC-A (5.6 ± 1.9) than for EDC-I (3.3 ± 0.7), C-i (2.5 ± 1.0) and C-i (2.6 ± 1.0) (p = 0.0001), while C-A values were in some cases similar to C-i and in others similar to EDC-i. No statistically significant difference was observed between the thirds (p > 0.05), except for EDC-i, which showed lower BS for the cervical (2.79 ± 0.46) compared to the apical third (3.8 ± 0.5), while the middle third in some cases had values similar to those of the apical and in others to the cervical third (3.2 ± 0.7) (p = 0.032). More mixed adhesive failures were found in the cervical third, and more adhesive failures to the sealer occurred in the middle and apical thirds (p = 0.014). A significant difference was observed between treatments in terms of adaptation of the adhesive interface, with a higher percentage of good adaptation using EDC (66.7%) than using C (40%), and a lower percentage of poor adaptation with EDC (10%) compared to C (20%) (p < 0.05).

**Conclusion:**

Root canal irrigation with EDC increased the longevity of the adhesive interface of an epoxy resin-based root-canal sealer.

The epoxy resin-based root-canal sealer AH Plus (Dentsply Sirona; Konstanz, Germany) is considered the gold standard among the endodontic sealers currently available on the market.^9^ This sealer stands out thanks to its adequate working time, good radiopacity, low dimensional change, low solubility, and flowability.^1^ Moreover, it chemically interacts with dentin through epoxy ring bonds to the amine groups of dentin collagen,^1^ which reduces marginal microleakage and favors the bond strength of the filling material to root dentin.^6^

The bond strength (BS) of endodontic sealer to root dentin is critical to maintain the sealing integrity of root canal fillings.^25,33^ During cleaning and shaping of the root canal, the mechanical action of the endodontic files associated with the chemical effect of the different irrigants can cause irreversible changes in the physical properties^23^ and chemical composition of the dentin tissue.^13^ It also results in the denaturation and solvation of the dentin collagen matrix.^2^

The dentin collagen matrix is a complex network composed of collagen fibrils, which play an essential role in the adhesion capacity between the resin-based endodontic sealers and dentin.^22^ The durability of the adhesive interface is directly related to the hydrophilic nature and organic structure of the endodontic sealer^33^ and the irrigation protocol used,^22^ which can directly interfere with the interaction between resin monomers and root dentin.^22^

Among the various commercially available irrigants, sodium hypochlorite (NaOCl) is the most widely used, due to its excellent antimicrobial spectrum, ability to dissolve both living and necrotic tissues, reduced toxicity at low concentrations,^14^ and efficacy in dissolving the organic component of the smear layer.^18^ However, one of the chemical reactions of NaOCl in the root canal that may negatively influence the adhesion of the sealer is related to residual NaOCl and its oxidative by-products, such as hypochlorite ions and hypochlorous acid, which can inhibit the free radicals produced during polymerization of methacrylate resins and compromise the adhesion of epoxy resin to the dentin walls.^26^

In fact, incomplete monomeric infiltration into the interfibrillar spaces may favor hydrolytic and enzymatic degradation of dentin collagen and make the adhesive interface potentially unstable, consequently causing a reduction in the longevity of the sealer bond strength.^20^ Thus, different synthetic substances with the potential to increase resistance to degradation and inhibit enzyme activity can be employed during the filling phase to expose the collagen network and promote intimate contact of the sealer with the root dentin.^11^ Ideally, this can contribute to maintaining the stability and longevity of the adhesive interface.^15^

Ethylenediaminetetraacetic acid (EDTA) is one of the most effective and well-studied chelating solutions in endodontics.^1^ EDTA is commonly used in association with NaOCl as a final intracanal surface treatment to optimize root canal cleaning,^32^ as it exposes dentin collagen due to its ability to chelate calcium ions, demineralize dentin, and remove the inorganic component of the smear layer.^30^ As a result, it promotes the opening of dentinal tubules and allows greater penetration of the sealer into the root dentin, consequently increasing the bond strength of the filling material to the root dentin.^14^

EDC (1-ethyl-3-[3-dimethylaminopropyl]carbodiimide hydrochloride) is a cross-linking agent that has demonstrated the ability to increase bond strength longevity^16^ by stimulating the formation of cross links which naturally exist between collagen molecules and fibrils; this makes dentin collagen more resistant to degradation,^28^ and can inactivate dentin metalloproteinases and cathepsins.^3^ In addition, EDC has also shown better results than other cross-linking agents in terms of biocompatibility and cytotoxicity. Previous studies have shown that the use of EDC at concentrations of 0.1, 0.3 and 0.5 M did not alter the response capacity of odontoblast-like cells,^27^ suggesting that EDC is a safe substance to be used as a final irrigant.^8,34^ Studies on the effect of cross-linking agents mostly show results related to the formation of a hybrid layer in coronal dentin, but few investigations have been done on the performance of those agents in root dentin,^16^ especially when associated with root-canal sealer. Hence, the effect of EDC as a final surface treatment after instrumentation of root canals and prior to obturation is still unknown.

Therefore, considering that the irrigants used during endodontic treatment may interfere with the bond strength of the endodontic sealers to root dentin,^14^ it is pertinent to evaluate the effect of EDC, a cross-linking inducing solution, on the bond strength longevity of AH Plus resin-based sealer (Dentsply Sirona; Konstanz, Germany) to dentin, as well as the quality of the adhesive interface formed between the filling material and root dentin surface. The null hypotheses tested were: (1) EDC does not interfere with the bond strength, (2) failure pattern, or (3) quality of the adhesive interface of the resin-based endodontic sealer to root dentin.

## Materials and Methods

After approval of the present study by the local research ethics committee (#40518920.0.0000.5419), sample calculation was performed. The t-test was performed using the Sigma Plot program (Systat Software; San Jose, CA, USA), considering a minimum difference of 1.0 MPa, standard deviation estimates of 0.75, statistical power of 0.8, and probability level of α = 0.05. The estimated number was 10 specimens per group.

### Sample Selection

Single-rooted maxillary canines recently extracted for periodontal reasons with a minimum root length of 17 mm, measured with a digital pachymeter (Digimess, Shiko Precision Gaging; Shandong, China) from the cementoenamel junction to the root apex, were obtained from the institution’s Human Tooth Biobank. The inclusion criteria were complete root formation, presence of a single canal, root and canal without curvature, no pulp-chamber calcifications, internal resorptions, or previous endodontic treatment. Twenty teeth were selected and kept in 0.1% thymol solution. Prior to the preparation of the samples, the teeth were washed in running water for 24 h and the external surface was cleaned with ultrasonic tips (Profi II Ceramic, Dabi Atlante; Ribeirão Preto, SP, Brazil).

### Sample Preparation

Subsequently, the crowns were sectioned in the buccolingual direction perpendicular to the long axis of the tooth near the cemento-dental junction, using an Isomet 1000 (Buehler; Lake Forest, IL, USA) saw with a 0.5-mm-thick diamond disk (South Bay Technology; San Clement, CA, USA) weighing 75 g, at a constant speed of 350 rpm under cooling, standardizing the roots at 17 mm.

Initially, the root canals were irrigated with 2 ml of 2.5% sodium hypochlorite (NaOCl) with a plastic syringe (Ultradent; South Jordan, UT, USA) and a 0.30-mm diameter NaviTip needle (Ultradent). The root canal was passively explored with a #15 K-file (Dentsply Sirona Endodontics; Ballaigues, Switzerland) until its tip was visible at the apical foramen, reaching the actual length of the tooth. From this measurement, 1.0 mm was subtracted to establish the working length (WL).

The biomechanical preparation was performed with a Reciproc 50.05 file (VDW; Munich, Germany) coupled to the counter-angle 6:1 Sirona (SN 25185, VDW) in the micromotor SMR 114058 (VDW), which was connected to the electric motor VDW Silver (VDW). The file was used passively, with pecking movements, and at every 3 advances it was removed from the canal and cleaned until it reached the WL. After each instrument removal, irrigation with 2 ml of 2.5% NaOCl was performed, followed by aspiration and flooding of the canals with a disposable plastic syringe and NaviTip needle.

### Final Irrigation Protocol

After instrumentation, the roots were randomly distributed into 2 groups (n = 10) according to the final irrigation protocol: 1. control group (C) – canal flooding with 17% EDTA for 5 min followed by irrigation with 5 ml of 2.5% NaOCl; 2. EDC group – canal flooding with 17% EDTA for 5 min followed by irrigation with 5 ml of 2.5% NaOCl and final surface treatment with 0.5 M EDC aqueous solution (ProteoChem; Denver, CO, USA) for 1 min. The 0.5 M EDC solution was manipulated by mixing 0.96 g of EDC (pH 6.0) and 10 ml of deionized water (pH 8.7). The solutions were applied using a disposable plastic syringe and NaviTip needle, and the canals were dried by aspiration with aspiration cannulas (capillary tips) and R50 absorbent paper cones (Reciproc, VDW).

### Sample Obturation

The adaptation of the gutta-percha cone R50 (Reciproc, VDW) at WL and obturation by the lateral condensation technique were verified by means of digital radiographs obtained in the orthogonal and mesioradial directions. AH Plus (Dentsply Sirona) was manipulated according to the manufacturer’s instructions and inserted into the root canal with a K-file #40 (Dentsply Sirona Endodontics) with counterclockwise rotating movements, after which the cone was greased with sealer and introduced with circular and gradual movement up to the WL. Excess filling material at the canal entrance was removed with a previously heated Paiva condenser (Golgram; São Caetano do Sul, SP, Brazil). Then, with the gutta-percha still plasticized, cold vertical condensation was performed, with light pressure in the apical direction for 5 s. The final cleaning of the root canal entrance was performed with a sponge dampened with alcohol, and the quality of the filling was checked by means of ortho- and mesioradial digital radiography. The specimens were stored in Eppendorf tubes and kept in 100% relative humidity at 37°C, remaining in these conditions for 72 h (equivalent to three times the sealer setting time).

### Slice Preparation

In order to obtain slices, the samples were placed on acrylic resin plates with the longitudinal axis parallel to their surface and fixed with hot glue, allowing the roots to be sectioned perpendicular to their long axis in the mesiodistal direction. A 0.3-mm-thick diamond disk (South Bay Technology; San Clement, CA, USA) under constant cooling at a speed of 350 rpm and weight of 75 g was used in an Isomet 1000 cutting machine (Lopes et al^16^). Three dentin slices of 1.0-mm (± 0.2 mm) thickness were obtained from each root third. The first two slices of each third were set aside for the push-out test, followed by the evaluation of the failure pattern, totaling six slices per root. One slice of each third was analyzed immediately after its procurement (n = 10), and the other slice was aged by storing at 37ºC in distilled water containing 0.4% sodium azide (renewed weekly) for 6 months (n = 10). The most apical slices from each third were analyzed with confocal laser scanning microscopy (n = 10).^16^

### Push-out Bond Strength Test

The slices were placed on a stainless-steel base with a 2.5-mm diameter hole in the central portion, which was attached to the lower portion of the Instron 25-19-106 universal testing machine (Instron; Canton, MA, USA). The slice was positioned on the metal base in the same direction as the hole with its cervical side facing down. This methodology ensured precise and reproducible alignment of the test, so that the rod used was centered on the filling material and not in contact with the dentin when the material was pressed and moved. Metal rods with active tips of 1.0-mm, 0.6-mm and 0.4-mm diameter were used, compatible with the diameter of the filling in the cervical, middle, and apical thirds, respectively. These rods were fixed in the upper portion of the testing machine and positioned over the intracanal filling material. The testing machine was driven with a constant speed of 0.5 mm/min until displacement.^16^

The compressive force required for displacement was measured in Newtons (N). To calculate the bond strength, the resulting force was converted into MPa by dividing it by the lateral area of the intracanal filling material. To calculate the lateral area adhered (SL), the geometric aspect of the filling material (sealer + gutta-percha) was considered. For this, before the test, the height (h) of each slice was measured with the aid of a digital pachymeter, and the radii (larger and smaller) were measured on images obtained using a Leica M165C stereomicroscope (Leica Mycrosystems; Wetzlar, Germany) with the aid of LAS v4.4 software (Leica Mycrosystems).

Thus, the adhesion area of the sealer (in mm^2^) was calculated by the lateral area (SL) formula:


$$\mathrm{SL}=\pi (R+r)\sqrt{h^{2}+(R-r{)}^{2}}$$

where R is the radius of the filling material in its coronal portion, r is the radius of the filling material in its apical portion and h is the height/thickness of the slice. From these data, the bond strength (BS) in MPa was calculated by dividing the force required to displace the gutta-percha by its lateral area (BS=F/SL).

### Failure Pattern

After the bond strength test, the type of failure was evaluated on images obtained by the Leica M165C stereomicroscope with a magnification of 10X to 25X. The failures were determined in percentages and classified according to the parameters established by Paiola et al^19^ as: a) adhesive to dentin, when the filling material debonded from the dentin; b) adhesive to the filling material, when the gutta-percha debonded from the sealer; c) mixed adhesive, when the gutta-percha debonded from both dentin and sealer; d) cohesive in dentin, when fracture occurred in the dentin; e) cohesive in the filling material, when fracture occurred in the filling material.

### Confocal Laser Scanning Microscopy (CLSM) of the Adhesive Interface

The third slice of each third (totaling 3 slices per specimen) of the adhesive interface was qualitatively and quantitatively evaluated of using CLSM. Initially, the surfaces of the slices were standardized with #600- and #1200-grit silicon-carbide papers under water cooling for 1 min each. Then, the surfaces were polished using a polishing machine (APL-4, Arotec; São Paulo, SP, Brazil) and felt disks with 0.3- and 0.05-μm alumina pastes (Arotec). Finally, the surfaces were conditioned with EDTA 17% for 10 s, washed with distilled water, and dried with absorbent paper towels.^12^

Then the specimens were positioned parallel to the table of the 3D confocal laser microscope (LEXT OLS4000, Olympus; Tokyo, Japan) to obtain images of the dentin-sealing material interface using the OLS4100 software (Olympus). Representative images of each slice were obtained with a 50X objective to evaluate the adaptation of the filling material. ImageJ software (NIH) was used to measure the gaps, performing 12 measurements at equidistant points at the adhesive interface from the root canal wall to the furthest point of the gaps.^12^

According to the methodology described in a previous study,^5^ the adaptation of the sealer to the root canal wall was classified according to the following criteria: a) good: most sections showed no gaps between sealer and dentin; b) acceptable: most sections showed some small gaps (<1 µm) between sealer and dentin; c) poor: most sections showed many gaps (between 1 and 10 µm) between sealer and dentin; d) no adaptation: most sections showed no adaptation between sealer and dentin (gaps >10 µm).

### Statistical Analysis

Bond strength data were expressed as means (± SD), evaluated for normality with the Shapiro-Wilk test (p > 0.05), and tested for homogeneity of variance using the Levene test (p > 0.05). Because the data showed normal distribution, the groups were compared using three-way ANOVA with Tukey’s post-hoc test to evaluate the factors “final irrigation protocol” (with or without EDC), “time of evaluation” (immediately and after 6 months), and “root third” (cervical, middle, and apical).

Fisher’s exact test (p < 0.05) was used to evaluate the association between the failure pattern after the BS test and the independent variables of the study (final irrigation protocol, time of evaluation, and root third). The Kruskal-Wallis test (p < 0.05) followed by the Dwass-Steel-Critchlow-Fligner post-test for multiple comparisons was used to analyze the data regarding the adaptation of the sealer to the dentin wall. The statistical tests were performed using the SAS 9.1 program (SAS; Cary, NC, USA) and based on a 5% significance level.

## Results

The three-factor ANOVA showed higher BS for EDC treatment after 6-month aging (5.6 ± 1.9 MPa) compared to the immediate assessment (3.3 ± 0.7 MPa), which was higher than the BS obtained in the immediate assessment of the control group (2.5 ± 1.0 MPa) (p = 0.0001) (Table 1). On the other hand, the BS obtained after 6 months in the control group (2.6 ± 1.0 MPa) (p = 0.0001) were in some cases similar to the immediate values of the control group, and in others to the immediate values of the EDC group (Table 1).

**Table 1:** Mean and standard deviation (in MPa) of the bond strength of the filling material to the root dentin in the cervical, middle, and apical thirds by study group

Root third	Dentin treatment			
	Control		EDC	
	Time of evaluation			
	Immediate	After aging*	Immediate	After aging
Cervical	2.5 ± 0.8^a^	2.3 ± 0.8^a^	2.8 ± 0.5^b^	4.4 ± 1.6^a^
Middle	2.8 ± 1.4^a^	2.8 ± 1.2^a^	3.2 ± 0.7^ab^	5.4 ± 1.7^a^
Apical	2.1 ± 0.5^a^	2.6 ± 1.0^a^	3.8 ± 0.5^a^	6.8 ± 1.7^a^
Total	2.5± 1.0^C^	2.6 ± 1.0^BC^	3.3 ± 0.7^B^	5.6 ± 1.9^A^

Superscript lowercase letters indicate statistically significant differences between lines (between thirds) and superscript upper case letters indicate statistically significant differences between columns (treatment x time of evaluation). *Aging consisted of storage for 6 months at 37ºC in distilled water containing 0.4% sodium azide.

No statistically significant differences were found between the root thirds (p > 0.05), with the exception of the EDC group in the immediate evaluation, in which lower BS was found for the cervical third (2.8 ± 0.5 MPa) than for the apical third (3.8 ± 0.5 MPa), while the middle third showed results similar in some cases to the apical third and in others to the cervical third (3.2 ± 0.7 MPa) (p = 0.032).

The failure pattern data for the dentin treatments at the different time points are presented in Table 2 in percent. Fisher’s exact test showed a difference for the variable root third (p = 0.014). The evaluation of the root thirds demonstrated a higher percentage of mixed adhesive failures for the cervical third than for the middle and apical thirds, which showed a higher percentage of adhesive failure to the filling material compared to the cervical third (p = 0.014).

**Table 2:** Type of failure after push-out test (percentages)

Failure type	Root third		
	Cervical	Middle	Apical
Ad	7.5	20.0	30.0
Ma	42.5	20.0	15.0
Af	42.5	57.5	55.0
Cd	2.5	0	0
Cf	5.0	2.5	0

Ad: adhesive to dentin; Ma: mixed adhesive; Af: adhesive to filling material; Cd: cohesive in dentin; Cf: cohesive in filling material.

Considering the analysis of the photomicrographs, the adaptation data of the filling material in percent are presented in Table 3. The Kruskal-Wallis test showed a statistically significant difference in the adhesive interface adaptation for the different dentin treatments (p = 0.026). The multiple comparison analysis (Dwass-Steel-Critchlow-Fligner test) showed that the group treated with EDC had a higher percentage of good adaptation (66.7%) compared to the control group (40%) (p < 0.05), as well as a lower percentage of poor (10%) and no adaptation (3.3%) compared to the control group (20% and 13.3% respectively) (Table 3).

**Table 3:** Quality of adaptation of the filling material to the root dentin (percentages)

Adaptation	Dentin treatment	
	Control	EDC
Good	40	66.7
Acceptable	26.7	20
Poor	20	10
No adaptation	13.3	3.3

**Fig 1:**
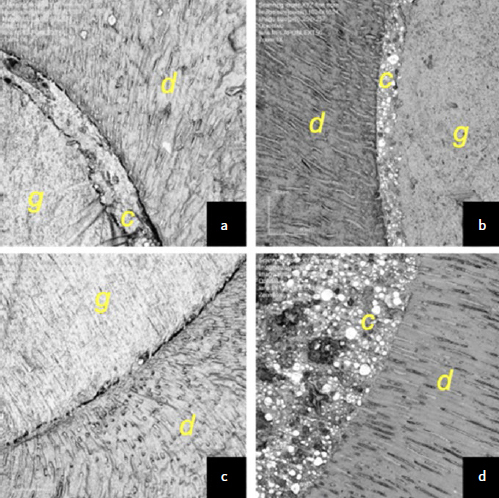
Photomicrographs of the adhesive interface formed between root dentin and filling material. (a) Adhesive interface of tooth treated with NaOCl + EDTA showing small gaps at the adhesive interface (50X); (b) adhesive interface of the tooth treated with NaOCl + EDTA + EDC showing regions of well-adapted adhesive interface (50X); (c) adhesive interface of tooth treated with NaOCl + EDTA showing small gaps at the adhesive interface (yellow asterisks) (50X); (d) adhesive interface of tooth treated with NaOCl + EDTA + EDC showing regions of well-adapted adhesive interface (50X). c: endodontic sealer; d: root dentin; g: filling material. Image colors have been changed to black and white.

Qualitative analysis of the CLSM images shows regions of gaps and voids at the interface between dentin and root filling material for the control group (Figs 1a and 1c). In the group treated with EDC, regions of gap-free adaptation were observed between the filling material and dentin (Fig 1d).

## Discussion

Despite the improvements in filling materials achieved in recent decades, degradation of the adhesive interface, premature decrease in bond strength, and consequent reduction in its durability may allow increased marginal infiltration^15^ and compromise the success of endodontic treatment. Thus, the present study evaluated the effect of final irrigation of the root canal with a cross-linking-inducing solution (EDC) on AH Plus bond strength, as well as the quality of the adhesive interface formed between the filling material and the root dentin.

Regarding the results of the push-out test, higher bond strength was found immediately and after 6 months for the group that received final surface treatment with EDC, compared to the control group. These results are probably related to the ability of EDC to react with the amino groups of the dentin collagen molecules,^35^ which leads to the formation of endogenous intermolecular and interfibrillar cross links and results in increased viscoelasticity, stability, and strength of the dentin matrix collagen.^28^ Moreover, EDC can inactivate the catalytic sites of proteolytic enzymes present in root dentin, such as matrix metalloproteinases (MMPs) and cysteine cathepsins.^10^ The change of the configuration of these catalytic sites or the allosteric inhibition of other modular domains that co-participate in dentin collagen degradation, such as glutamate and aspartate residues, promotes increased collagen stiffness and inhibition of proteolytic activity.^35^ This explains the higher bond strength and the long-term adhesive interface stability.^28^ Therefore, the first null hypothesis, that EDC does not interfere with bond strength, should be rejected.

The favorable influence on bond strength promoted by EDC treatment is confirmed by the CLSM analysis of the adhesive interface: the different surface treatments showed statistically significant differences in the adaptation of the filling material to the root dentin (Table 3). The use of EDC as the final irrigant resulted in a well-adapted adhesive interface characterized by the absence of gaps and voids, whereas EDTA resulted in small gaps at the adhesive interface (Fig 1). Thus, the third null hypothesis, that EDC does not influence the quality of the root-sealer-dentin adhesive interface, should also be rejected.

Considering the failure pattern analysis, in general, a predominance of mixed adhesive failures was found in the cervical third, while the middle and apical thirds exhibited more adhesive failures to the filling material. These findings corroborate with previous studies,^7,12^ which demonstrates that the filling materials were able to mechanically adhere to dentin walls,^24^ suggesting that both final treatments promoted the adhesion of AH Plus. Thus, the second null hypothesis, that EDC does not interfere with the failure pattern, should be accepted.

Regarding the methodology, it is important to highlight that a push-out test was used, which is considered a reliable method for evaluating the bond strength to root dentin by allowing the force to be applied parallel to the adhesive interface, simulating clinical conditions,^31^ besides enabling the determination of the bond strength in the different root canal thirds.^17^ For this, metal bases and rods with active tip and holes of compatible diameters for each third were used to favor the application of force and the distribution of shear stresses as close as possible to the adhesive interface formed between the sealer and the root dentin.^36^ Complementarily, the failure patterns were examined using stereomicroscopy after the push-out test, wherease the adaptation of the sealer to the root dentin was evaluated by means of confocal laser scanning microscopy. This equipment allowed the acquisition of photomicrographs at different magnifications,^34^ which enabled the measurement of gaps by quadrants,^5^ followed by classification and statistical analysis by means of scores.^4^

To evaluate the longevity of bond strength, accelerated aging was performed by direct exposure of the slices to distilled water containing 0.4% sodium azide, since root sectioning before storage in water leads to rapid diffusion through the adhesive interface, resulting in its rapid hydrolytic degradation.^29^ Although storing the slices in water for 6 months and subsequently performing the push-out test cannot reproduce the clinical aging conditions and functional loads, this method makes it possible to evaluate the effect of metalloproteinase-inhibiting agents on the stability of the adhesive interface.^29^ This method has been used in studies that seek to determine the longevity of the bond strength of fiberglass posts to root dentin.^37^

Given the results of the present study, it can be stated that the final irrigation of root dentin with EDC prior to filling with AH Pus was a valid alternative to increase the longevity of the bond strength of the adhesive interface. However, it is emphasized that more in-vitro studies are needed to evaluate the bond strength and adaptation of the adhesive interface for longer periods, in addition to clinical studies to verify the behavior of this material in the oral environment.

## Conclusion

The use of EDC cross linker makes the adhesive interface more stable by stimulating the formation of cross links between collagen fibrils, making the dentin collagen more resistant to the degradation process. Therefore, it can be concluded that final irrigation of the root canal with EDC increases the longevity and quality of the adhesive interface of the epoxy resin-based root-canal sealer.

## Clinical relevance

Dentin pretreatment with EDC resulted in higher bond strength and better adaptation of the adhesive interface.
